# Vaginal Birth after Cesarean Section in Taiwan: A Population-Based Study

**DOI:** 10.3390/jcm8081203

**Published:** 2019-08-12

**Authors:** Yung-Hsiang Ying, George Linn, Koyin Chang

**Affiliations:** 1 College of Management, National Taiwan Normal University, Taipei 116, Taiwan; 2 Department of Obstetrics and Gynecology, Show Chwan Memorial Hospital, Changhua City 500, Taiwan; 3 Department of Healthcare Information and Management, Ming Chuan University, Taoyuan 333, Taiwan

**Keywords:** population-based study, hierarchical analysis, health policies, cesarean section

## Abstract

The rate of vaginal birth after cesarean section (VBAC) is extremely low in Taiwan probably due to the high perceived risk of trial of labor after a cesarean (TOLAC). To promote the benefits associated with vaginal birth, this study provides evidence to potentially assist relevant public authorities adopt appropriate guidelines or optimize health insurance reimbursement policies to achieve a higher VBAC rate. Employing the National Health Insurance (NHI) Claim Data, this study analyzes women’s adoptions of birth-giving methods for those who had previous cesarean section (CS) experiences. Empirical methods include logit, probit, and hierarchical regression models controlling women’s demographics, incentive indicators, as well as hospital and obstetrician characteristics. Taiwan continues to have a decreasing trend in VBAC rate even with an increase in NHI payment for vaginal birth delivery in 2005, which stimulated a surge in VBAC rate only temporarily. Factors that significantly influence women’s adoption of VBAC include institution-specific random effects, weekend admission, comorbidities during pregnancy, and income and fertility of women. Change in service payment from National Health Insurance (NHI) to healthcare providers constitutes an effective policy in directing clinical practices in the short term. Constant and systematic policy review should be undertaken to promote safe and beneficial medical practices. The results of the study suggest that women’s adoption of birth-giving method is dominated by non-medical considerations. Significant institution-specific effects imply that women might not be well-informed regarding their optimal birth-giving choice. Health education and training programs for hospital personnel should be kept up to date to better serve society.

## 1. Introduction

The proportion of women giving birth by cesarean section (CS) in Taiwan reached 37% in 2016, the second-highest globally after Italy at 39% [1,2]. The reasons for women adopting cesarean delivery include medical considerations, such as complications during pregnancy or labor and delivery processes, and non-medical ones, such as fear of pain or more predictable delivery date [3,4]. In Chinese culture, it is particularly common to have babies delivered at certain “auspicious” points in time. Many Chinese believe that the positive destiny of a person is crucially determined by the time and date of that person’s birth. Obstetricians might also discourage women who have had a previous CS from choosing vaginal birth to avoid risks. [5] Relative to the vaginal birth after cesarean section (VBAC) rates of 8.5–30% in the U.S. [6] and 30–50% in European countries, [7] Taiwan’s low rate of VBAC of only 1.5–4.0% in recent decades [8,9,10] implies that it is pervasive to view VBAC as a hazardous practice. However, the incidences surrounding uterine rupture occur in less than 1% of all attempts [11,12,13,14]. Trial of labor after a cesarean (TOLAC) should be encouraged because there are major benefits associated with vaginal birth, such as more rapid recoveries, potentially fewer hazards in future pregnancies, [15,16,17] and less likelihood of childhood diseases, including asthma, obesity, and allergies [18,19]. As a result, the increasing rate of primary and repeat cesarean delivery is a topic of concern for many clinicians and patients.

Women and obstetricians, in general, view TOLAC as a relatively perilous birthing practice, which might be attributed to underinvestment in obstetric departments in healthcare institutions. The low rate of birth in this country (the crude birth rate decreased from 2.46 in 1981 to 1.18 in 2015) [20] has caused obstetrics to remain a relatively non-lucrative specialty, and thus has not attracted great attention regarding investment in equipment and infrastructure. To promote more vaginal birth and decrease the usage of CS, the Ministry of National Health Insurance (MNHI) in Taiwan amended the payment rate of vaginal birth to be the same as CS in May 2005. It is expected that the policy change will potentially affect the rate of vaginal birth, as well as the rate of VBAC.

Guidelines of American College of Obstetricians and Gynecologist (ACOG) advise that TOLAC is most safely undertaken in hospitals where staff can immediately perform an emergency cesarean delivery [21]. Certain institutional factors, such as hospital layout and the availability of supporting teams in the delivery room, are also important [22]. Understanding the factors that influence the adoption rate of VBAC offers practical implications. The results of our study are pertinent for the amendment of relevant policies to facilitate complication-free deliveries. They are also of interest to an international interdisciplinary audience, as they provide insight into the appropriate choice of delivery method based on institutional infrastructure and women’s personal traits in a country with a distinct culture from its Western counterparts.

## 2. Data and Empirical Methods

### 2.1. Variables and Data Sources

The data adopted in this study, the Longitudinal Health Insurance Dataset 2010 (LHID2010), were obtained from the Taiwanese National Health Research Institutes (NHRI), which provided data from National Health Insurance launched in March 1995. It constitutes one of the largest nationwide population-based databases in the world, and many scientific studies have utilized its data. Four subsets of data were retrieved from LHID2010. The “Monthly Claims Summary for Inpatient Claims” (claims dataset) comprised medical claims data for one million randomly-sampled patients from all NHI program beneficiaries from 1996 to 2011. The “Registry for Beneficiaries’ (ID) data sets contained registration and demographic data for those one million sampled patients. Primary diagnoses for each admission in the NHIRD database were assigned a code based on the International Classification of Diseases, Ninth Revision, i.e., an ICD-9 code. Data regarding institutional characteristics and physician characteristics were obtained from HOSB and PER datasets, respectively. For a more detailed description of the database and its accuracy level, please see Ying (2017). The description of the variables and the datasets they were collected from are presented in Table 1.

This research extracts all women who had past birth-giving experiences at least two times from the one million sampled patients in the time period of 1996 to 2011. ICD-9 for each birth case was examined to identify the type of deliveries: 650–659 for vaginal birth and 669.7 for CS. The type of birth deliveries from the second time or above were then classified into one of the following four categories:

(1) Vaginal birth after previous CS experience (VBAC)
(2) Vaginal birth after previous vaginal birth experience (VBAV)
(3) CS after previous CS experience or elective repeat cesarean surgery (ERCS)
(4) CS after previous vaginal birth experience.

Our aim is to determine whether there are potential non-medical factors that influence birth- giving methods for women who gave birth a second time or more. Among the four categories, the first three constitute our research interests; the fourth one is excluded from our study since women experienced vaginal birth prior to CS, implying that medical reasons instead of personal preferences dominated the decision-making process. The second category, VBAV, is not CS-related but retained in our sample pool as a baseline for comparison. We are investigating the determinants for VBAC to take place, controlling for demographic variables, such as age, income status and residential location, as well as physicians’ characteristics, such as age and previous experiences. Institutional tiers that imply the resourcefulness and properness of infrastructures are also controlled. Other necessary influential factors are women’s complications or comorbidity conditions during pregnancy and delivery. According to the Office on Women’s Health, ref. [23] commonly seen complications (ICD-9 codes) include malposition and malpresentation of fetus (652), disproportion in pregnancy labor and delivery (653), abnormality of organs, and soft tissues of pelvis (654), long labor (662), forceps/vacuum extractor delivery, delivered (669.51), cesarean delivery without indication delivered with or without antepartum condition (669.70), cesarean delivery without indication delivered with or without antepartum condition (669.70), complicated delivery or labor (669.9), and other known or suspected fetal and placental problems affecting management of mother (656).

Complications or comorbidity conditions during pregnancy or the birth-giving process are recorded as a dummy variable to reflect a potential need for CS. The details of the variables employed in the research are summarized in Table 1.

Even though our data extend back to 1996, the analysis of the study only starts from 2001 because earlier data lacked completed demographic information, and the number of birth-giving experiences in the past could not be traced for the earlier years. Although our data from 1996 to 2000 will be included when extracting women’s past birth-giving experiences, birth cases will not be analyzed for these years. By doing so, possible bias from missing information can be minimized.

### 2.2. Empirical Methods

Three research methods are employed for analysis. Since the variable of interest is whether or not VBAC takes place for a particular birth case, logit and probit methods are appropriate since the dependent variables are binary choices. Multiple level (hierarchical) random effect regressions are also utilized to specially control for correlations between observations within the same categories under different hierarchical levels.

### 2.3. Ethics Approval and Consent to Participate

The study protocol was reviewed and approved by the institutional review board of a medical center in Taiwan, Chen-Hsing Hospital, with the approval number (380)120-27. This manuscript does not contain any individual person’s data. Thus, consent to participate is deemed unnecessary according to national regulations This manuscript does not contain any individual person’s data. Thus, consent for publication is not applicable. The data on which the conclusions of the manuscript rely are available upon request. Please email koyin@kchang.net for detailed information.

### 2.4. Logit and Probit

The regression model of logit or probit takes the following form:

Birth Choice_it_ = F(β_0_ + β_1_X_it_ + β_2_P_t_ + β_3_T_i_ + ε_it_) (1)

where birth method = 1 if it is VBAC and 0 if it is VBAV or ERCS for birth case i in year t; F is the functional form with a normal distribution for the probit model [24] and a logistic distribution for the logit model; [25] X_it_ is the explanatory variable for women’s characteristics, including age, income, comorbidities/complications, fertility (number of births) and location, and the other control variables, including obstetricians’ and hospitals’ characteristics and whether or not it is a weekend admission; P is a dummy variable that refers to the 2005 reimbursement policy change for vaginal birth; T represents a time trend that captures the underlying trends of VBAC or CS; and ε is the residual with assumed ε ~ N(0, 1).

### 2.5. Multiple-Level Hierarchical Regression

It is plausible to assume that the incidence of VBAC does not follow a random pattern, but rather is clustered in better-equipped institutions where managerial attitude is more supportive of the practice. Thus, the incidence can be structured hierarchically with different levels [26]. Observations under the same structural level may share similarities due to cultural, techonological, managerial, or even preference differences. As women’s babies are delivered by the same obstetricans, in the same hospitals, and within the same districts, correlations may exist. Thus, hierarchical regression methods are applied to control for potential bias associated with the correlations. Three levels are divided in this study: (1) district level (based on the zip code of women’s residences); (2) institution level (tier); and (3) patient level for analysis. Obstetricians are not included as a hierarchical level since too many different physicians were involved in the sample. Our regression model is then constructed as follows:

Birth Choice_ijkt_ = F(β_0_ + β_1_X_ijkt_ + β_2_P_t_ + β_2_T + ψ_jt_ + γ_jkt_ + ε_ijkt_) (2)

where X_ijkt_ is a vector of covariates; and β is a corresponding vector of parameter estimates. The vector of covariates includes a constant with explanatory variables measured at the different levels. The random effects for institution j, district k, and individual i are, respectively, ψ_jt_ [N(0, σ_ψ_^2^)], γ_jkt_ [N(0, σ_μ_^2^)], and ε_ijkt_ [N(0, σ_ε_^2^)]. Details of the theoretical explanation of the hierarchical model can be found in Treno et al. (2003) and Gelman et al. (2006) [27,28].

## 3. Results

The statistical summary of our sampled cases is presented in Table 2. The rate of VBAC is calculated for only women who had previous cesarean section (CS) experiences. The highest rate was 7.1% in 2007, and lowest in 2010 with only 4.4%. The average age in our sample is higher than the average age of birth-giving women since our sample includes only women who had at least one previous birth experience. Although income is not directly available from NHIRD, insurance premiums are calculated based on a sliding scale from personal income. Thus, the income status used in this research can accurately reflect the income level of the insured. Complication describes the rate of women who had comorbidities or complications during pregnancy or the delivery process. The highest rate is 35.2%, and occurred in 2009.

The results of the three regression models are presented in Table 3. Probit and logit are in columns 1 and 2, respectively. The results of the hierarchical logit regression are shown in columns 3 and 4; the former one regressed with only two levels (patient and institution) and the latter one with three levels (patient, institution, and district). Among the results in all models, policy, income status, institution tier, and fertility are all statistically significant in determining the usage of VBAC. Specifically, women from wealthier families are less likely to undertake VBAC, while older women and women with higher fertility are more likely to undertake VBAC. Deliveries that take place in medical centers are more likely to be VBAC than in other institution types. Policy change also has a strong impact on birth choice in all models, indicating that financial incentive is important in the decision-making process.

In terms of institution classification (tier), medical centers provide more VBAC relative to regional hospitals, district hospitals, and private clinics. Whether or not women experienced medical complications during pregnancies or the delivery process has an impact on the usage of delivery method in the probit and logit models. However, this effect is not salient in hierarchical models. Other prominent determinants include the age of obstetricians and weekend admission. Institution random effect is significant at the 1% level in the hierarchical model, while district effect is not significant.

## 4. Discussion

Applying probit, logit, and hierarchical logit models, this research reveals that many factors play significant roles in the adoption of childbirth delivery method. The medical condition of pregnant women, measured by the presence of comorbidity or complications during pregnancy and the delivery process, only significantly influence the decision when estimating with probit and logit models. This variable is not significant in the random effect model, indicating that unobserved institution-specific characteristics are related to women’s health condition, which influences which tiers of healthcare provider they chose for giving birth. Women might self-select to give birth in better-equipped hospitals if their perceived health condition is not as good. As a result, the health condition of women is not a significant determinant in the random effect hierarchical model.

**Figure 1:**
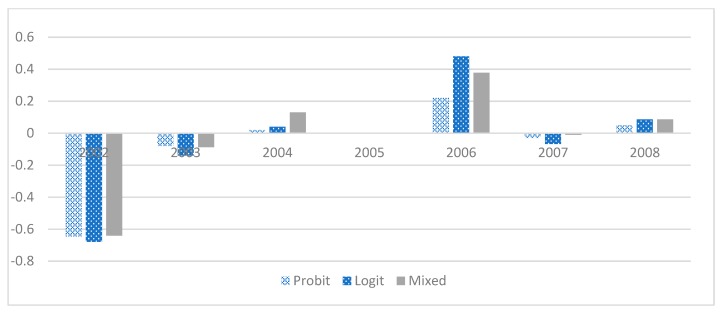
The effect of the 2005 policy change on the probability of VBAC. Note: Y axis shows coefficients from different regression models. In the Probit model, coefficients represent probability of using natural delivery. In the logit and mixed models, coefficients represent log odds ratio.

For women who have higher fertility, it is more likely for them to adopt VBAC. The result indicates that women gain more knowledge about the benefits of vaginal birth (or the cost of CS) through personal experiences. The usage of VBAC becomes a more natural choice of birth method when fertility rate is increased, holding other variables constant. When women are admitted to a hospital or clinic during the weekend, VBAC is more likely than for non-weekend admissions. Two plausible explanations for this phenomenon are: (1) since family members tend to be freer during the weekends, they give more support to the women to wait for vaginal birth than those who admitted during workdays; and (2) obstetricians may be more willing to try vaginal birth if labor has progressed for the cases of emergency delivery since there might be fewer supporting personnel on duty in the operation room during the weekends. The policy regarding the change of insurance payment has been widely studied in the past [29,30,31,32]. Our finding suggests a positive influence on birth- giving decision at the 1% level of significance in all models, which is not in accordance with previous findings when caesarean rate was studied. Thus, for a closer examination, we apply a similar approach to Chandra, [33] and the same regression analyses are performed again with the additional inclusion of a dynamic pattern of effects for each of the three years prior to and after the policy change; the results are shown in Table 4 and Figure 1. Although not all years exhibit significant results, a general pattern of negative coefficients before the policy change and positive coefficients after the policy change can be discerned. The positive effect in 2006 is particularly prominent, implying that at least a short-term upward surge was stimulated by the policy change. Two plausible reasons for the different policy impact from this study compared to the extant literature are: (1) previous research focuses on CS, and not VBAC; and (2) this study analyzes the likelihood of each case instead of the incidence rate across the population, as was done previously. Thus, the new evidence could shed light on the impact of the incentive policy on child delivery choice.

Another interesting finding of this study is the negative relationship between the likelihood of VBAC and income. Traditionally, it has been believed that CS is regarded as a superior delivery method among wealthier families. One possible reason for this could be that it allows parents to deliver babies at a specific auspicious time and date. Certain superstitious thinking remains pervasive in Chinese culture, and it is especially important for families of a higher economic status. The rate of VBAC peaked in 2007, two years after the policy change. However, the long-term trend of VBAC is decreasing, as can be seen from the negative coefficients of time trend in Table 3. Our reported VBAC rate is higher than that in previous findings, which may be calculated based on entire birth number, instead of only women who had CS experiences, as done in this study. However, the VBAC rate is greatly lower than that of Western countries, implying that women are misinformed to a certain extent about potential hazards associated with VBAC. It is critical that women be given a range of choices and information about the benefits of vaginal birth. Vaginal delivery should be a clear option for women to consider, including those who have previous CS experiences. By doing so, women’s and babies’ health can be promoted. Healthcare costs can also be reduced since vaginal birth, in general, is a more cost-effective method than CS.

### Limitations

This study employs the National Health Insurance database. As such, it shares any drawbacks of these insurance claim data, including that the severity level of comorbidity is unavailable and income status of the insured is presumptive. In addition, birth-giving records are extracted from the one million insured database for a total of a 16-year time span. The records of the women giving birth prior to 1996 are not included. Those who gave birth for the second time after 1996 may have been misperceived as the first time. This might cause VBAC to be underestimated. However, the underestimation is only quite serious for data in the earlier years. Omitting observations from those years, and only considering observations after 2001, would help to minimize the problem in future research.

## 5. Conclusions

Taiwan is considered to be a country with a first-rate healthcare system. The low rate of VBAC is unusual, which might be a result of underinvestment in hospital delivery rooms due to the declining birth rate in Taiwan. In addition, the payment rate for vaginal delivery is lower than cesarean section (CS) prior to 2005, and obstetricians were not incentivized to provide options for women, especially for those who had previous CS experiences.

Our results show that institutional factors and incentive policies are the key determinants for VBAC to be undertaken, even when women’s characteristics and comorbidity are controlled. The impacts are statistically significant in the probit, logit, and random effect hierarchical logit models. In addition, institutional effects are salient while district effects are not in the hierarchical logit model, implying that managerial attitude and infrastructural capacity of the institutions constitute the fundamental drivers of the differences. Since insurance payment policy is another clear driving force to increase VBAC rate, adjustment of the payment rate can be further evaluated. Under the current policy, the Taiwan MNHI provides the same remuneration to providers for vaginal birth and CS without consideration of women’s previous birth experiences. Adequate policy change is advised by including the birth-giving history of the women when classifying payment categories. Moreover, relevant education should also be provided to healthcare personnel to help them understand or create safety standards for VBAC. In conclusion, this study suggests that a woman’s birth-giving method is dominated by non-medical considerations, which include institutional and physician characteristics. Given our data constraints, we can only infer that VBAC is more likely to take place in medical centers in which there are more support staff and better equipment. Further empirical research is critically needed to understand the details about how different characteristics of healthcare providers impact birth methods.

## Figures and Tables

**Table 1:** Definition of Variables and Data Sources.

Variable	Definition	Data Source
VBAC	Vaginal birth after cesarean section	Claim data
Policy	Dummy variable indicating the payment increase in May 2005 that approximately doubles the NHI payment of vaginal birth delivery.	
Age	Age of women when giving birth.	ID data
Income status ^a^	In NTD (000).	ID data
Complication	Presence of complications or comorbidities during pregnancies.	Claim data
Conglomerate	Baby is delivered in a hospital owned by a business conglomerates.	HOSB
Public	Baby is delivered in a government-owned hospital.	HOSB
Religion	Baby is delivered in a hospital with a religious affiliation.	HOSB
Clinic	Baby is delivered in a private-clinic setting.	HOSB
Doc_Age	Age of the delivering obstetrician.	PER data
Female	Baby is delivered by a female obstetrician.	ID data
Fertility	Previous number of births of the women.	ID data
Doc_Vol	Number of babies delivered by the obstetrician within the year.	PER data
Weekend_adm	Women admitted into hospitals/clinics over weekends.	Claim data

Note: ^a^ Income status is imputed from the insurance premium by MNHI, which is consistently underestimated. NTD, New Taiwan dollars.

**Table 2:** Statistical Summary.

	2001	2002	2003	2004	2005	2006	2007	2008	2009	2010	2011	Total
**VBAC**	0.063	0.047	0.078	0.087	0.090	0.098	0.065	0.071	0.059	0.044	0.053	0.068
	(0.24)	(0.21)	(0.26)	(0.28)	(0.28)	(0.29)	(0.25)	(0.26)	(0.24)	(0.21)	(0.22)	(0.25)
**Age**	28.85	29.02	29.40	29.73	30.01	30.21	30.55	30.81	31.33	31.79	32.00	29.99
	(4.40)	(4.43)	(4.41)	(4.45)	(4.46)	(4.52)	(4.52)	(4.49)	(4.38)	(4.36)	(4.28)	(4.57)
**Income** **(‘000)**	18.16	18.51	22.31	22.86	23.69	23.60	24.67	24.44	25.15	25.95	26.95	22.50
	(10.3)	(11.4)	(11.4)	(12.5)	(14.2)	(13.8)	(14.7)	(14.8)	(15.3)	(16.6)	(17.4)	(13.8)
**Complication**	0.316	0.327	0.335	0.314	0.316	0.332	0.337	0.327	0.352	0.349	0.348	0.331
	(0.47)	(0.47)	(0.47)	(0.46)	(0.47)	(0.47)	(0.47)	(0.47)	(0.48)	(0.48)	(0.48)	(0.47)
**Conglomerate**	0.243	0.245	0.230	0.250	0.257	0.275	0.290	0.274	0.283	0.282	0.298	0.266
	(0.43)	(0.43)	(0.42)	(0.43)	(0.44)	(0.45)	(0.45)	(0.45)	(0.45)	(0.45)	(0.46)	(0.44)
**Public**	0.087	0.095	0.082	0.077	0.083	0.072	0.072	0.074	0.067	0.083	0.068	0.081
	(0.28)	(0.29)	(0.28)	(0.27)	(0.28)	(0.26)	(0.26)	(0.26)	(0.25)	(0.28)	(0.25)	(0.27)
**Religion**	0.031	0.027	0.030	0.030	0.026	0.030	0.030	0.029	0.031	0.027	0.031	0.030
	(0.17)	(0.16)	(0.17)	(0.17)	(0.16)	(0.17)	(0.17)	(0.17)	(0.17)	(0.16)	(0.17)	(0.17)
**Clinic**	0.331	0.334	0.354	0.336	0.336	0.322	0.294	0.313	0.320	0.295	0.298	0.321
	(0.47)	(0.47)	(0.48)	(0.47)	(0.47)	(0.47)	(0.46)	(0.46)	(0.47)	(0.46)	(0.46)	(0.47)
**Doc_Age**	42.70	43.06	43.8	44.31	44.90	45.22	45.76	46.32	46.88	47.47	47.96	44.75
	(5.67)	(5.54)	(5.59)	(5.97)	(6.05)	(6.08)	(6.27)	(6.39)	(6.39)	(6.66)	(6.56)	(6.33)
**Female**	0.062	0.065	0.067	0.069	0.079	0.080	0.088	0.093	0.092	0.108	0.107	0.079
	(0.24)	(0.25)	(0.25)	(0.25)	(0.27)	(0.27)	(0.28)	(0.29)	(0.29)	(0.31)	(0.31)	(0.27)
**Fertility**	2.103	2.145	2.161	2.189	2.202	2.223	2.226	2.241	2.230	2.247	2.244	2.175
	(0.31)	(0.37)	(0.41)	(0.44)	(0.46)	(0.50)	(0.49)	(0.54)	(0.53)	(0.56)	(0.56)	(0.45)

Note: VBAC is the rate among women who had previous cesarean experience. Standard deviations are in parentheses.

**Table 3:** Determinants of VBAC: Analysis Results from Different Models.

	Probit	Logit	Mixed I	Mixed II
**Policy**	0.26 ***	0.596 **	0.445 ***	0.740 ***
	(2.31)	(2.24)	(2.99)	(5.37)
**Patient Characteristics**				
**Age**	0.017 *	0.037 *	0.021 *	0.021 *
	(2.31)	(2.39)	(2.33)	(2.22)
**Income**	−0.093 ***	−0.209 ***	−0.102 ***	−0.115 ***
	(−3.67)	(−3.14)	(−3.32)	(−2.88)
**Complications**	−0.211 ***	−0.543 ***	−0.075	−0.107
	(−3.36)	3.90)	(−0.92)	(−1.28)
**Fertility**	0.393 ***	0.797 ***	0.613 ***	0.631 ***
	(7.38)	(7.15)	(10.08)	(10.03)
**Institution Classification**				
**Medical center**	0.290 *	0.608 *	0.573 *	0.471 **
	(2.54)	(2.51)	(2.36)	(2.92)
**Clinic**	−0.775	−1.784	−0.547	−0.784
	(−1.47)	(−1.54)	(−0.73)	(−1.07)
**Regional_H**	−0.055	−0.121	0.213	−0.006
	(−0.55)	(−0.56)	−1.14	(−0.04)
**Market Share**	−0.418	−0.795	−0.82	−0.162
	(−0.72)	(−0.64)	(−0.99)	(−0.19)
**Institution Ownership**				
**Conglomerate**	−0.168	−0.349	−0.325	−0.308 *
	(−1.65)	(−1.60)	(−1.39)	(−3.01)
**Public**	−0.102	−0.224	−0.221	−0.184
	(−0.78)	(−0.81)	(−0.85)	(−0.99)
**Religion**	0.289	0.614	0.466	0.4
	(1.51)	(1.51)	(1.06)	(1.47)
**Districts**				
**Taipei**	0.179	0.378	0.155	0.219
	(0.83)	(0.81)	(0.50)	(0.66)
**Central**	−0.128	−0.258	−0.037	−0.169
	(−0.50)	(−0.46)	(−0.10)	(−0.43)
**East**	0.087	0.169	−0.02	0.142
	−0.33	−0.29	(−0.05)	−0.36
**Kao-ping**	0.482 *	1.006 *	0.391	0.591
	(2.20)	(2.13)	(1.23)	(1.77)
**South**	0.139	0.287	0.177	0.185
	(0.66)	(0.63)	(0.59)	(0.57)
**Doc. Characteristics**				
**Ob_age**	0.012 **	0.026 **	0.013 *	0.012 *
	(2.32)	(2.37)	(1.74)	(1.81)
**Female**	−0.018	−0.025	0.033	−0.073
	(−0.15)	(−0.10)	(0.20)	(−0.47)
**Doc. Vol**	0.003	0.006	0.001	0.005
	(0.80)	(0.75)	(0.15)	(0.93)
**Other factors**				
**Weekend Adm.**	0.155 *	0.324 *	0.201 *	0.204 *
	(2.46)	(2.44)	(2.40)	(2.44)
**Time Trend**	−0.065 ***	−0.130 ***	−0.096 ***	−0.000 ***
	(−3.39)	(−3.18)	(−3.51)	(−3.04)
**Random Effect**				
**Zip Code**				0.00
				(0.17)
**Institution**			0.82 ***	1.02 ***
			(11.07)	(8.09)
**Constant**	146.710 ***	308.606 ***	191.084 ***	194.128 ***
	(4.84)	(4.83)	(4.69)	(4.68)
**Obs no.**	34,914	34,914	34,914	34,882
**Chi2**	145.59	150.84	185.575	202.32
**AIC**	6754.82	6747.45	6912.39	7041.78
**BIC**	6966.33	6958.97	7123.91	7261.73

Note: The omitted reference for institution classification is “district hospital”. The omitted region is the “North”. Market share is the Herfindahl-Hirschman index calculated based on the number of babies delivered in the year. Coefficients for logit and mixed models are log odds ratios. Numbers in parentheses are *z*-values. *, **, *** represent the 0.10, 0.05, and 0.01 statistical significance level, respectively.

**Table 4:** Dynamic Model of Regression Analyses.

	Probit	Logit	Mixed Model	Likelihood
**2002**	−0.645 ***	−1.139 ***	−0.77 **	−0.64 **
	(−3.73)	(−3.66)	(−4.27)	(−4.68)
**2003**	−0.086	−0.151	−0.09	−0.087
	(−0.60)	(−0.58)	(−0.54)	(−0.65)
**2004**	0.026	0.044	0.13	0.13
	(−0.22)	(−0.2)	(0.95)	(0.95)
**2006**	0.226 **	0.397 **	0.319 **	0.377 **
	(2.07)	(2.01)	(2.44)	(2.44)
**2007**	−0.038	−0.071	−0.097	−0.009
	(−0.31)	(−0.33)	(−0.07)	(−0.07)
**2008**	0.055	0.083	0.087	0.08
	(0.42)	(0.36)	(0.61)	(0.60)
**Chi**	192.846	180.525	55,741.766	
***p* value**	0.00	0.00	0.00	
**AIC**	5800.953	5804.472	5997.446	
**BIC**	6040.738	6044.257	6237.198	

Note: Omitted year for baseline comparison is 2005, when the NHI payment policy was changed. Numbers in parentheses are z-values. Likelihoods are estimated from odds ratio based on the results from the mixed model. Likelihoods estimated by the logit model generate similar results; thus, they are not reported due to space considerations. Full regression results are not reported since all three models generate comparable results as in Table 3. **, *** represent the 0.05, and 0.01 statistical significance level, respectively.

## Abbreviations

ACOG	American College of Obstetricians and Gynecologists
CS	cesarean section
ERCS	elective repeat cesarean surgery
LHID2010	Longitudinal Health Insurance Dataset 2010
MNHI	Ministry of National Health Insurance
NHIP	National Health Insurance Program
TOLAC	trial of labor after a cesarean
VBAC	vaginal birth after cesarean section
VBAV	vaginal birth after previous vaginal birth
